# Transcriptomic analysis reveals a tissue-specific loss of identity during ageing and cancer

**DOI:** 10.1186/s12864-023-09756-w

**Published:** 2023-10-26

**Authors:** Gabriel Arantes dos Santos, Kasit Chatsirisupachai, Roberto A. Avelar, João Pedro de Magalhães

**Affiliations:** 1 Laboratory of Medical Investigation (LIM55), Urology Department, Faculdade de Medicina FMUSP, Universidade de Sao Paulo, Sao Paulo, Brazil; 2 Genomics of Ageing and Rejuvenation Lab, Institute of Inflammation and Ageing, University of Birmingham, Birmingham, B15 2WB UK; 3 Institute of Life Course and Medical Sciences, University of Liverpool, Liverpool, L7 8TX UK

**Keywords:** Functional genomics, Geriatric oncology, Geroscience, Oncogenomics

## Abstract

**Introduction:**

Understanding changes in cell identity in cancer and ageing is of great importance. In this work, we analyzed how gene expression changes in human tissues are associated with tissue specificity during cancer and ageing using transcriptome data from TCGA and GTEx.

**Results:**

We found significant downregulation of tissue-specific genes during ageing in 40% of the tissues analyzed, which suggests loss of tissue identity with age. For most cancer types, we have noted a consistent pattern of downregulation in genes that are specific to the tissue from which the tumor originated. Moreover, we observed in cancer an activation of genes not usually expressed in the tissue of origin as well as an upregulation of genes specific to other tissues. These patterns in cancer were associated with patient survival. The age of the patient, however, did not influence these patterns.

**Conclusion:**

We identified loss of cellular identity in 40% of the tissues analysed during human ageing, and a clear pattern in cancer, where during tumorigenesis cells express genes specific to other organs while suppressing the expression of genes from their original tissue. The loss of cellular identity observed in cancer is associated with prognosis and is not influenced by age, suggesting that it is a crucial stage in carcinogenesis.

**Supplementary Information:**

The online version contains supplementary material available at 10.1186/s12864-023-09756-w.

## Introduction

Tissues are formed by the combinations of cells expressing different transcripts and proteins, which shape their morphology and function [1]. Differences between tissues are driven by transcriptomic programs and signatures, which change during normal organismal development and in detrimental processes such as ageing and various diseases [2, 3].

One hypothesis in gerontology is that tissues lose their cellular identity during ageing, which contributes to age-related dysfunctions. Although recent studies support this idea for some tissues, we still do not have enough evidence to confirm this hypothesis, which highlights an important topic for the field [4–7].

Cancer cells are known to gain plasticity and stemness during tumor initiation and progression, and recently, unlocking phenotypic plasticity has been considered a “new” cancer hallmark [8, 9]. Ageing is one of the main risk factors for most cancers, which may link tissue specificity processes, the onset of cancer and progressive age-related disruptions [10]. Moreover, cancers of different origins have different behaviour and development, and the relationship between the tumor and the original tissue needs to be better elucidated to improve our understanding of cancer biology and find better treatment options [11–13]. Finally, growing evidence confirms that the molecular landscape of cancers from old and young patients are different, but we do not yet know whether patterns of tissue-specific identity influence these differences in any way [14].

In this study, we explore the relationship between genes differentially expressed in cancer and ageing with tissue-specific identity, using data from TCGA (The Cancer Genome Atlas), a comprehensive consortium that uses thousands of cancer samples to try to decipher the tumour landscape through sequencing and clinical data, and GTEx (Genotype-Tissue Expression), a public resource to study tissue-specific gene expression and regulation from healthy samples. Our results show evidence of a downregulation of tissue-specific genes in most cancers and in aged human tissues.

## Methods

### Data acquisition and processing

The mRNA expression data in read counts from the TCGA harmonized data (data aligned to hg38) and clinical data (XML files) were downloaded using TCGAbiolinks (version 2.14.1) [15], as described in Chatsirisupachai et al. [16].

RNA-Seq-based gene expression data from normal tissues (version 8) were downloaded in read counts from the GTEx portal (https://gtexportal.org) [17]. According to GTEx documents, the sequencing reads were aligned to the human reference genome GRCH38/hg38.

First, we separated the TCGA or GTEx data according to cancer/tissue, and then we removed genes with less than 1 count in more than 30% of samples. TCGA and GTEx analyses were done independently. We used the biomaRt package to keep only the protein-coding genes in all analyses [18].

### Genes differentially expressed in ageing (ageing-DEGs)

GTEx data was used to find differentially expressed genes with age. Samples without complete information were filtered out, and tissues with less than 50 complete samples were also excluded. GTEx only lists age ranges (i.e., 20–29, 30–39, 40–49, 50–59, 60–69 and 70), so age was approximated to 25, 35, 45, 55, 65 and 75, respectively, as before [3]. The sample numbers for each tissue used in this study are shown in table Supplementary File 1. We follow the RNA-seq analysis workflow of Law et al. as described below [19].

GTEx GENCODE gene IDs were converted to Ensembl gene IDs using the cleanid() function from the grex package version 1.9 [20]. After ID conversion, 18 Ensembl IDs were duplicated – these genes were removed from the read counts.

Samples were grouped into their respective tissues and processed together.

First, to correct for library size variation between samples, the trimmed mean of M-values (TMM) normalisation method was applied using the calcNormFactors() function with default parameters [21]. Then, read counts were first converted to counts per million (CPM) to identify and exclude genes with low expression, using the cpm() function with default parameters from the edgeR package[22–24]. Counts were then voom transformed to adjust for heteroscedasticity using the voom() function with default parameters [25].

For each tissue, fold change with age was calculated using the model below. If any variable is not present in the tissue (e.g., sex for prostate or region for blood), it is disregarded in the analysis. All information on the subjects was taken directly from the GTEx portal (https://gtexportal.org/home/datasets).

$${\text{Y}}_{\text{i}\text{j}}={\alpha }{\text{A}\text{g}\text{e}}_{\text{i}}+{\beta }{\text{S}\text{e}\text{x}}_{\text{i}}+{\gamma }{\text{D}\text{e}\text{a}\text{t}\text{h}}_{\text{i}}+{\delta }{\text{R}\text{e}\text{g}\text{i}\text{o}\text{n}}_{\text{i}}+{{\epsilon }}_{ij}$$

The variables are defined as follows:

- *Y*_*ij*_: The expression level of gene *j* in sample *i.*
- *Age*_*i*_: The age of sample *i* – continuous variable.
- *Sex*_*i*_: The sex of sample *i –* categorical variable.
- *Death*_*i*_: The death classification of sample *i* based on the 4-point Hardy scale *–* categorical variable [26].
- *Region*_*i*_: The tissue region cells were extracted from for sample *i –* categorical variable.
- ε_*ij*_: The error term for gene *i* in sample *j.*

Linear models were generated using the R package limma, using the lmFit() function with default parameters[27, 28]. Genes were considered DEGs if they matched the following criteria: (i) The p-values derived from the empirical Bayes moderated t-statistics were less than 0.05 after Benjamini-Hochberg (BH) FDR correction; and (ii) the absolute log2 (fold change) across 50 years of age (from 25 to 75), represented as 50* log2 (fold change) was greater than log2(1.5) [3].

### Genes differentially expressed in cancer (cancer-DEGs)

Of all the cancers available in TCGA, we selected for our analyses only organs with at least ten samples of adjacent normal tissue available. The selected TCGA projects are: BLCA, BRCA, COAD, ESCA, HNSC, KICH, KIRC, KIRP, LIHC, LUAD, LUSC, PRAD, READ, STAD, THCA and UCEC. For BRCA, we kept only samples from female patients. The sample number and definition for each organ (cancer and normal samples) are shown in table Supplementary File 1.

To generate a list of differentially expressed genes in cancer (cancer-DEGs), we compared tumour samples with adjacent normal tissue samples. Data were processed and analyzed using the R package DESeq2 [29] using default parameters. A gene was considered differentially expressed when Fold Change > 2 and FDR < 0.01. The p-value adjustment was made using BH methodology.

### Classification of genes based on specificity category

First, we need to classify all genes according to their tissue specificity. For this, we downloaded the Tau Index-based classification by Daniel Palmer et al. [30]. Briefly, the tau index is based on GTEx data and indicates how specific or widely expressed a gene is, with tau = 1 indicating expression specific to only one tissue and tau = 0 indicating similar expression in all tissues.

We subsequently established four specificity categories, further divided into two groups. The initial group, termed ‘Pan-tissue categories,‘ exclusively relies on tau values. As a result, genes in this group remain the same regardless of the tissues analyzed. The second group, called ‘Tissue-Specific categories,‘ incorporates tau values and tissue-specific average expression. Consequently, the genes in this group are different according to the tissue analysed. The categories are shown below:

- Pan-tissue categories:

“High Tissue Specificity genes” = Tau > 0.8;

“Low Tissue Specificity genes” = Tau < 0.2.

- Tissue-Specific categories:

“Tissue-Specific genes” = Tau > 0.95 and average expression > 1 in the tissue of interest;

“Tissue-Unexpressed genes” = Expression = 0 in the tissue of interest.

It is important to note that the Pan-tissue categories define how specific the expression of a gene is without specifying in which organ it is being expressed. On the other hand, Tissue-specific categories are based on the expression (or lack of expression) of genes only in the tissue being analyzed. We kept only protein-coding genes and excluded transcripts where tau = NA (i.e., that are not expressed in any tissue).

The values of the “Pan-tissue” group are based on the original paper by Palmer et al. [30]. In the “Tissue-specific” group, we tried to be as strict as possible to ensure tissue specificity, so we used extreme tau values. The “Tissue-Specific” category, we used tau > 0.95 because using tau = 1 would generate insufficient genes for further analysis, but we guaranteed tissue specificity and consistent expression using only genes with average expression above 1. Here it is important to note that although some authors consider “Tissue-Specific genes” to be genes expressed only in one tissue, we use a broader classification, in which a tissue-specific gene has higher expression in one tissue but is expressed in one or a few tissues, an approach applied in several papers [31–35].

The Tau data and average tissue expression used in this study is in table Supplementary File 2. The numbers of genes in each category and the background list (all remaining genes in the tau classification) are shown in Table 1.

**Table 1:** Number of DEGs in TCGA and GTEx samples and number of genes on tissue specificity categories

High Tissue Specificity Genes	4851					
Low Tissue Specificity Genes	3464					
Genes in the background	17,836					
	**Up_Ageing**	**Down_Ageing**	**Up_Cancer**	**Down_Cancer**	**Tissue-unexpressed**	**Tissue-specific**
Breast - BRCA	154	39	1802	1379	3833	35
Colon - COAD	80	1013	1630	1710	3861	73
Esophagus - ESCA	82	70	1145	840	3958	37
Kidney - KICH	0	0	1848	1997	4238	113
Kidney - KIRC	0	0	2214	1269	4238	113
Kidney - KIRP	0	0	1809	1358	4238	113
Liver - LIHC	14	21	1476	882	5872	162
Lung - LUAD	235	226	1919	1518	3473	117
Lung - LUSC	235	226	2627	2176	3473	117
Prostate - PRAD	1000	872	608	1097	3492	29
Stomach - STAD	21	13	1065	1591	4195	46
Thyroid - THCA	84	35	1167	641	3764	78
Uterus - UCEC	647	917	2364	1769	4122	27
BLCA			1415	1450	3928	44
HNSC			1290	1361		
READ			1737	1748		
Adipose Tissue	174	163			4223	27
Adrenal Gland	311	121			4736	56
Blood	236	96			5988	126
Blood Vessel	596	149			4481	25
Brain	347	343			3899	356
Heart	73	33			5483	51
Muscle	232	89			6289	83
Nerve	256	331			3934	77
Ovary	340	382			4303	42
Pancreas	54	18			5610	67
Pituitary	6	3			3382	242
Salivary Gland	887	432			3899	94
Skin	9	33			3874	195
Small Intestine	218	583			3688	119
Spleen	24	12			4277	119
Testis	99	25			1961	1248
Vagina	71	31			1588	46

*“Genes in the background” are all protein-coding genes, which are used as the background list in contingency analyses (i.e., overlap analyses); **“Up” and “Down” represent whether genes are upregulated or downregulated

### Overlap analyses

We performed a contingency analysis (i.e., overlap analysis) by overlapping the DEGs (differentially expressed genes) related to cancer and ageing with the four categories using basic R functions. The overlap was considered significant if FDR < 0.05 (Fisher’s exact test followed by Benjamini-Hochberg correction).

To make sure that the pattern found in the previous analysis was biologically accurate, we used an alternative specificity classification and repeated the overlaps with the same parameters. For this, we downloaded data from Uhlén et al., where the authors’ classified genes based on RNA expression in a tissue-specific manner [36]. Briefly, in Uhlén et al., genes are divided into six main categories: “Tissue enriched”, “Tissue enhanced”, “Group enriched”, “Expressed in all”, “Mixed” and “Not Detected”. We then adapted these categories to our study, where we have the following “alternative categories” (number of genes and background list in each category in table Supplementary File 3:

- Pan-tissue categories:

High Tissue specificity genes = “Tissue enriched”, “Tissue enhanced” and “Group enriched”;

Low Tissue specificity genes = “Expressed in all” and “Mixed”,

- Tissue-Specific categories:

Tissue-Specific genes = “Tissue enriched”, “Tissue enhanced” or “Group enriched” in the tissue of interest.

Tissue-unexpressed = FPKM < 1 in the tissue of interest.

It is important to note that in Palmer’s classification, HNSC and READ data could not be analyzed in a tissue-specific manner as we do not have data available for the corresponding normal tissue (tonsil and rectum). In the alternative classification, BRCA, UCEC, blood, blood vessel, breast, nerve, pituitary, uterus, and vagina face the same limitation since their respective tissues are unavailable.

### Gene ontology enrichment analysis

For the enrichment analyses, we used the webtool WebGestalt (https://www.webgestalt.org/) [37] with the following parameters: Over-Representation Analysis (ORA) and Gene Ontology (Biological Process non-redundant). The reference list is the human protein-coding genome. Only significant results (FDR < 0.05) were included.

For High and Low Tissue Specificity genes we used as input all genes in these categories. For cancer-specific analyses, we used as input the downregulated-DEGs that are Tissue-Specific genes, or upregulated-DEGs that are Tissue-Unexpressed genes.

### Survival analyses

For this analysis, we selected only the genes that lie in the overlap between the cancer-DEG and one of the four categories. We use the expression signature of those genes to construct overall and disease-free survival curves. All survival analyses were performed on GEPIA2 (http://gepia2.cancer-pku.cn/#survival) using the median expression of the signature to segregate the two groups [38]. The constructed heatmap was based on the log2 hazard ratio (Mantel-Cox test), and the result was considered significant when FDR < 0.1.

### Cancer analyses based on patients’ age

To analyze whether the patient’s age influenced the loss of tissue identity in cancer, we separated the TCGA samples into two groups: young and old. The young group comprises 30% younger samples and the old group 30% older samples. We kept only cancers with at least ten control samples in each normal group (old and young normal). To avoid confounding factors, we compared the two groups regarding the T pathological stage (Fisher’s exact test); if the p-value < 0.05, we considered the groups different and excluded this cancer from the subsequent analysis (Figure S1). So, for this analysis, we keep the following TCGA projects: KIRP, HNSC, COAD, LIHC, LUSC, LUAD, and BRCA. The age distribution of the two groups is shown in figure S2.

The differential expression and overlap analysis were done as before. To compare the expression (Fold Change) between the two groups, genes that lie in the overlap between the cancer-DEG and one of the four categories in each group were compared by Mann-Whitney U test using GraphPad Prism 8.0, and the difference was considered significant when FDR < 0.05.

## Results

### Differentially expressed genes in cancer and ageing

We first identified cancer-DEGs (table Supplementary File 4) and ageing-DEGs (table Supplementary File 5) in 26 human tissues and 16 cancer types, respectively. Moreover, we classified all the genes according to their tissue specificity based on tau’s categories, a metric of tissue-specificity described in Palmer et al. [30] (table Supplementary File 6). The numbers of cancer- and ageing-DEGs as well as genes classified according to tissue specificity are shown in Table 1 for the cancers and tissues analyzed.

It is important to note that we employ two types of gene tissue-specificity: (1) tissue-specific and tissue-unexpressed genes are classified based on the expression signature within a particular tissue being analyzed. For example, if analyzing the liver, we consider tissue-specific and tissue-unexpressed genes in the liver. On the other hand, (2) pan-tissue genes, whether high or low, indicate the broad level of tissue-specific expression across all tissues. A high specificity gene is expressed only in one or a few tissues, while a low specificity gene is expressed similarly across most tissues.

To provide insights into the functions of High and Low Tissue Specificity genes, used in all analyses regardless of tissues, we performed functional enrichment analyses (Supplementary File 7). As expected, in general, the low specificity genes are associated with basic cellular metabolism, while the high specificity genes have more specialized functions.

**Fig. 1:**
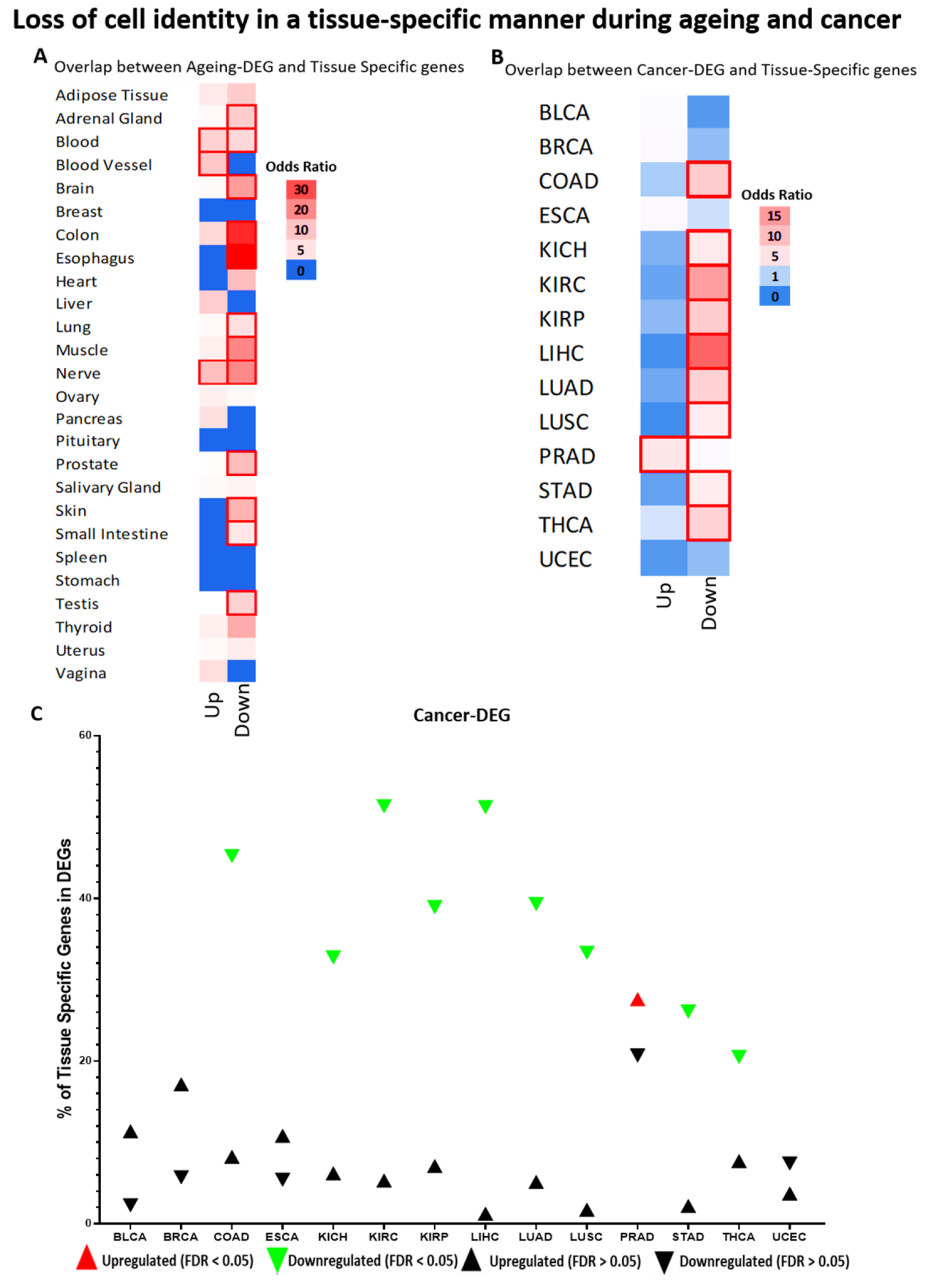
Overlap between DEGs and Tissue-Specific genes in ageing (**A**) and cancer (**B, C**). **A-B** Heatmap of odds ratio on the chance of the overlap. Red borders represent significant results (FDR < 0.05). A Ageing-DEGs, B Cancer-DEGs. “Up” and “Down” represent whether genes are upregulated or downregulated. C Percentages of tissue specific-genes for each tissue that are differentially expressed in cancer. Each triangle represents the percentage of genes and the direction of expression. Coloured triangles are the statistically significant results

**Fig. 2:**
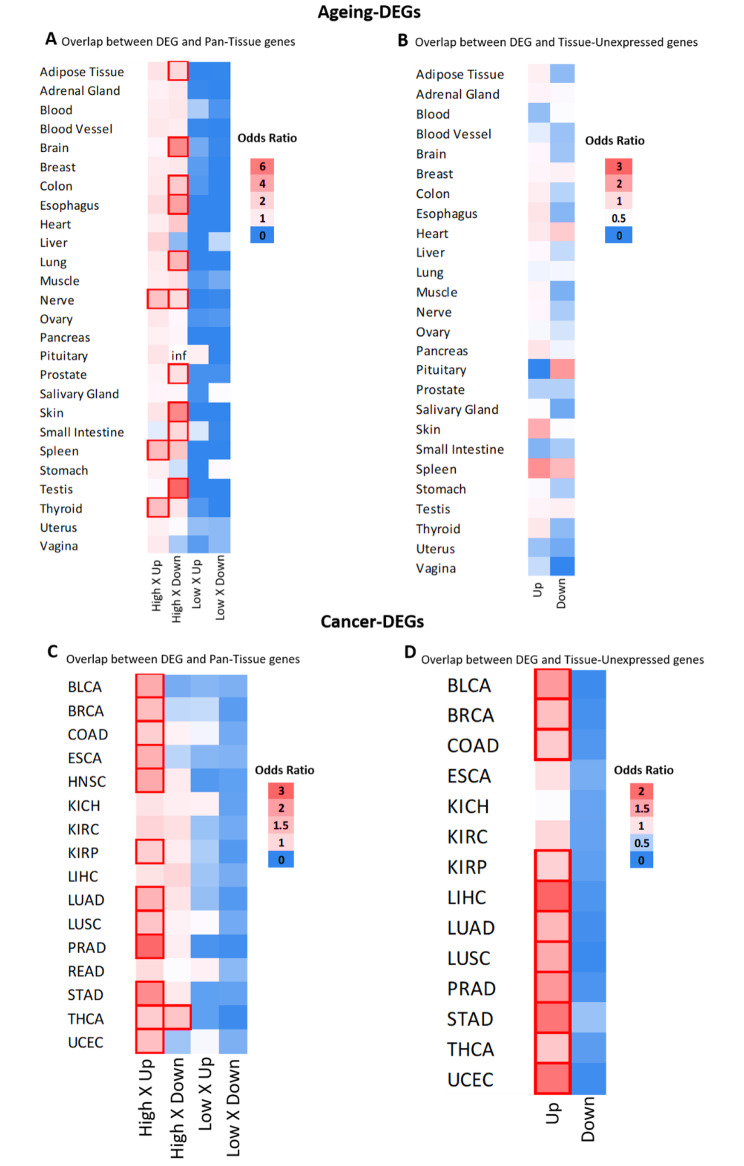
Overlap between DEGs and tissue specificity categories. Heatmap of odds ratio on the chance of the overlap. Red borders represent significant results (FDR < 0.05). **A-B** Ageing-DEGs; **C-D** Cancer-DEGs. Inf = Odds ratio tends to infinity due to the low number of downregulated DEGs, but the result is not significant. “Up” and “Down” represent whether genes are upregulated or downregulated. “High” an “Low” represents Pan-Tissue group of genes with overall high or low tissue-specific expression across tissues

We then investigated the relationship between ageing-DEG (Fig. 1A) and cancer-DEG (Fig. 1B and C) with tissue-specific genes. Afterwards, we overlapped ageing-DEG (Fig. 2A and B) and cancer-DEG (Fig. 2 C and D) with the pan-tissue genes and tissue-unexpressed genes. In the following sections, we describe how these results impact ageing and cancer, respectively.

### Loss of cellular identity in ageing observed in 40% of tissues analyzed

We overlapped the ageing-DEGs with the Tissue-specific category (Fig. 1A) and observed a pattern of tissue-specific loss of identity during ageing. Of the tissues analyzed, around 40% show enrichment of downregulated Tissue-specific genes (Fig. 1A), with some exceptions. Adrenal gland, brain, colon, esophagus, lung, muscle, prostate, skin, small intestine, and testis present downregulation of Tissue-specific genes, without presenting significant results in the opposite direction. Validating these results, we observed similar patterns in genes with High Tissue specificity (Fig. 2A). No significant results were identified for Tissue-unexpressed genes (Fig. 2B).

These results suggest that, although we have a trend of loss of tissue identity for some tissues with ageing, it is not a global phenomenon, presenting some exceptions. One possible explanation for this is that shifts in gene expression during ageing are more subtle and hence more difficult to detect.

### A robust pattern of cellular identity loss is observed in most cancers studied

Repeating the same approach as before, we overlay the cancer-DEGs with the four specificity categories and observe a pattern in most of the cancers analyzed. First, we observed an enrichment of downregulated DEGs in Tissue-specific genes (Fig. 1B). At the same time, we can see a significant number of upregulated DEGs in High Tissue specificity and Tissue-unexpressed (Fig. 2C-D, respectively).

It is essential to highlight that the results of Tissue-specific genes (Fig. 1B) are the most relevant, as opposed to the overexpression of genes from other organs and typically inactive genes (Fig. 2C-D), which is expected considering the nature of cancer. We show in Fig. 1C the percentage of Tissue-Specific genes that are cancer-DEGs, highlighting the results that were statistically significant from the previous analysis. Considering the statistically significant results, we observe an enrichment of tissue-specific genes downregulated in 9 cancers, ranging from 20.5% in THCA to 51.3% in KIRC.

These results make biological sense and are in line with the literature. Considering the pan-tissue group, our analyses showed cancers commonly overexpress genes associated with specific functions of other organs and tissues (High Tissue specificity genes). In a tissue-specific manner, they activate genes usually unexpressed and downregulate genes typically highly expressed in the original tissue. THCA (Fig. 2C) and PRAD (Fig. 1B and C) are the only significant exceptions to this pattern.

Furthermore, as we found a trend of overexpression of High Tissue Specificity genes, we tried to answer which healthy tissues these genes are typically expressed in, repeating the same approach but overlapping only the upregulated cancer-DEGs with the Tissue-specific genes from all GTEx tissues. As shown in Figure S3, we found no obvious pattern, which is aligned with the notion of generalized genetic instability in cancer cells.

Afterwards, to provide biological context to the observed patterns, we performed cancer-specific functional enrichment analyses for the downregulated Tissue-Specific DEGs (Figure S4) and the upregulated Tissue-Unexpressed DEGs (Supplementary File 8).

Regarding the downregulated Tissue-Specific DEGs in BLCA, BRCA, ESCA, LUAD, PRAD, THCA, and UCEC, no significant results were obtained (FDR < 0.05). Despite the limitations, some tissue-specific functions were observed to be downregulated in cancer. For instance, we noted a downregulation of genes associated with digestion in COAD and STAD, respiratory gaseous exchange in LUSC, and organic anion transport in the three renal tumors (KICH, KIRC, and KIRP) (Figure S4).

As for the upregulated Tissue-Unexpressed DEGs, significant results were found in all cancers except for THCA (FDR < 0.05). We identified more than 140 terms associated with at least one cancer, and in general, we observed the activation of genes associated with cellular proliferation, DNA metabolism, immune response, embryogenesis, and morphogenesis (Supplementary File 8).

Finally, to further validate our findings and ensure their biological accuracy, we conducted additional analyses using an alternative classification system for tissue-specific genes (Figure S5). This alternative classification was based on data from Uhlén et al. [36], where genes were categorized into groups based on their RNA expression patterns in different tissues. We adapted these groups into our specificity categories to create alternative gene classification (the number of genes and the background list for each category can be found in Supplementary File 3). The biggest difference between the two analyses is in the results of the pan-tissue categories (Figures S5 and D). This is probably because the alternative categories are much less stringent, which results in many more genes and consequently more significant results. However, results from Tissue-specific group are quite similar (Figures S5B, C, E and F), indicating that the observed pattern is biologically relevant.

### Loss of tissue-identity is associated with cancer prognosis

**Fig. 3:**
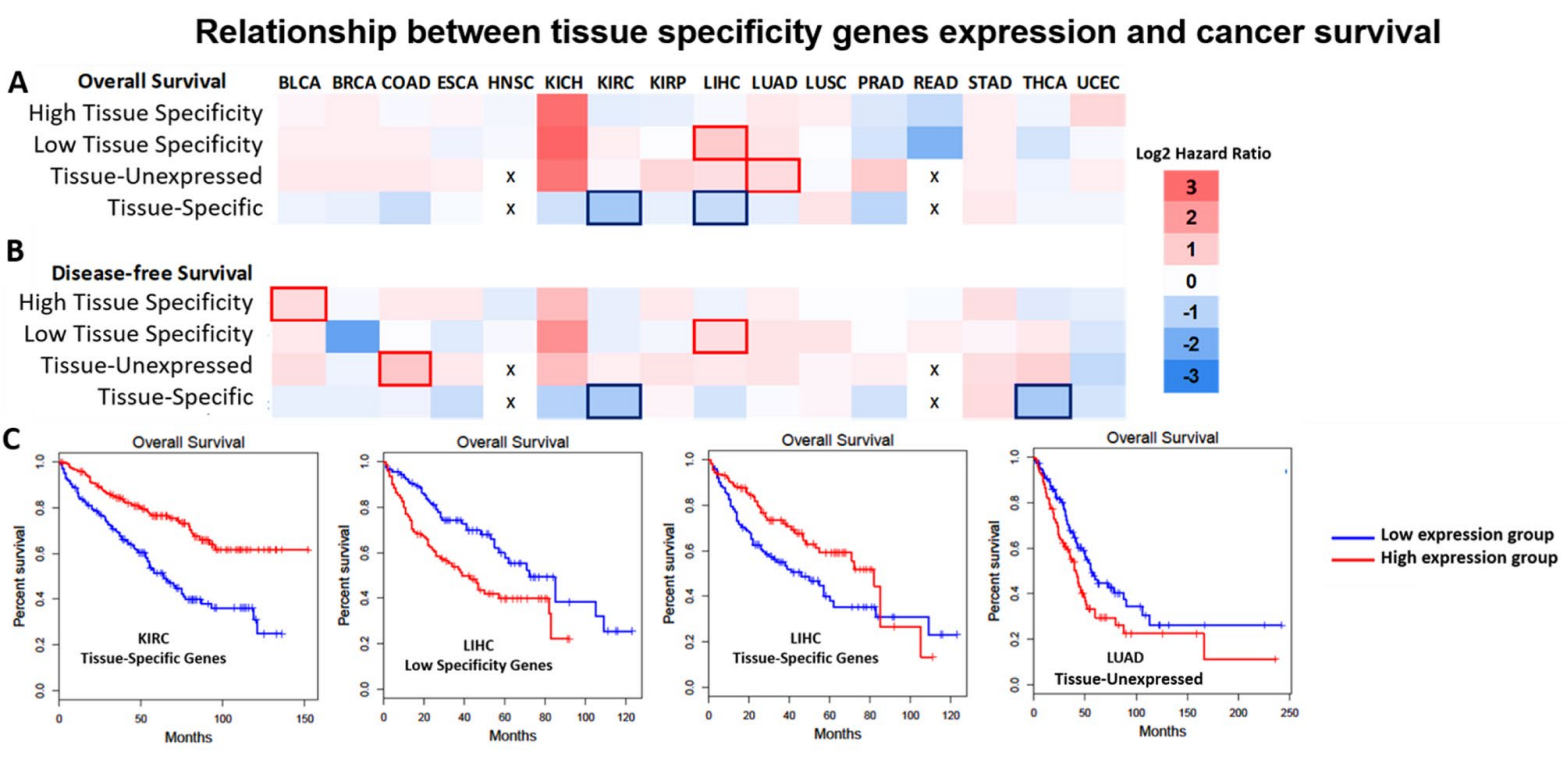
Relationship between tissue specificity genes and cancer survival. **A** and **B** Heat map of hazard ratio of overall and disease-free survival, respectively, statistically significant results (Mantel-Cox test, FDR < 0.1) are highlighted with blue or red borders, according to the direction of the expression signature and the worst survival. The x represents where analysis cannot be done in a tissue-specific manner. **C**) Kaplan-Meier curves of the significant overall survival results from the previous heatmap

After identifying the pattern of loss of tissue specificity in cancer, we sought to understand whether this impacts patient survival. To do this, we used the genes in the overlap between cancer-DEG and one of the four categories of tau specificity and built an expression signature. Using the median of expression as a cutoff, we constructed overall survival and disease-free survival analysis comparing the high and low expression signature groups (Fig. 3). Looking at the heatmaps (Fig. 3A and B), we can see a trend in the Tissue-specific group. We can observe that most cancers show a positive hazard ratio pattern (i.e., high expression group associated with the worst survival) in the Tissue-unexpressed genes, and oppositely, a negative hazard ratio pattern (i.e., low expression group associated with the worst survival) in Tissue-specific genes.

When analyzing the curves of the significant results (Fig. 3C and Figure S6), we observe that almost all observations align with the pattern observed previously: The upregulation of High Tissue specificity or Tissue-unexpressed is associated with worse survival, and the downregulation of Tissue-specific genes is associated with worse prognosis. A partial exception is observed in LIHC, where the overexpression of Low tissue specificity genes is related to a worsening in overall and disease-free survival. Considering these results, we suggest a trend for the loss of tissue identity to increase cancer aggressiveness.

### Age does not influence the loss of tissue specificity in cancer

Previous studies demonstrated that there are important molecular differences when considering the age of cancer patients [16, 39–41]. Then, considering that perhaps the loss of tissue identity occurs in ageing, we tested the hypothesis that the patient’s age influences the pattern of expression of specificity genes in cancer. For this, we separated the TCGA cancers into two groups in relation to age, as described in the methods, and generated two lists of DEGs for each tumor.

The old cancer-DEGs were obtained by directly comparing the 30% oldest cancer samples against the 30% oldest non-cancer tissue samples. The young cancer-DEGs were obtained in the same way, but using the 30% youngest samples. These genes are in Tables Supplementary File 9 and Supplementary File 10.

We then analyzed the overlap between the DEGs of the two groups (Table 2) and observed that most differentially expressed genes are shared independent of patient age, but a few hundred genes are unique to the old or young group. Besides that, 5 genes showed opposite expression patterns: COX4I2 in HNSC; NR4A2 and NR4A1 in COAD; and CYP26A1 and FDCSP in BRCA. It would be interesting to explore whether these genes are important in differentiating cancers from old and young patients, but this analysis is beyond the scope of this study.

**Table 2:** Overlap between old and young cancer-DEGs

Cancer	Up-Old and Young Shared	Down-Old and Young Shared	Up-Old Exclusive	Down-Old Exclusive	Up-Young Exclusive	Down-Young Exclusive
**KIRP**	1176	777	210	220	784	434
**HNSC**	866	670	310	324	323	604
**COAD**	1406	1276	393	391	183	138
**LIHC**	1108	553	226	156	523	335
**LUSC**	2301	1837	247	338	424	236
**LUAD**	1616	1132	170	354	438	314
**BRCA**	1484	1110	568	514	441	158
**Total**	9957	7355	2124	2297	3116	2219

*“Up” and “Down” represent whether genes are upregulated or downregulated in cancer in young or old patients

**Fig. 4:**
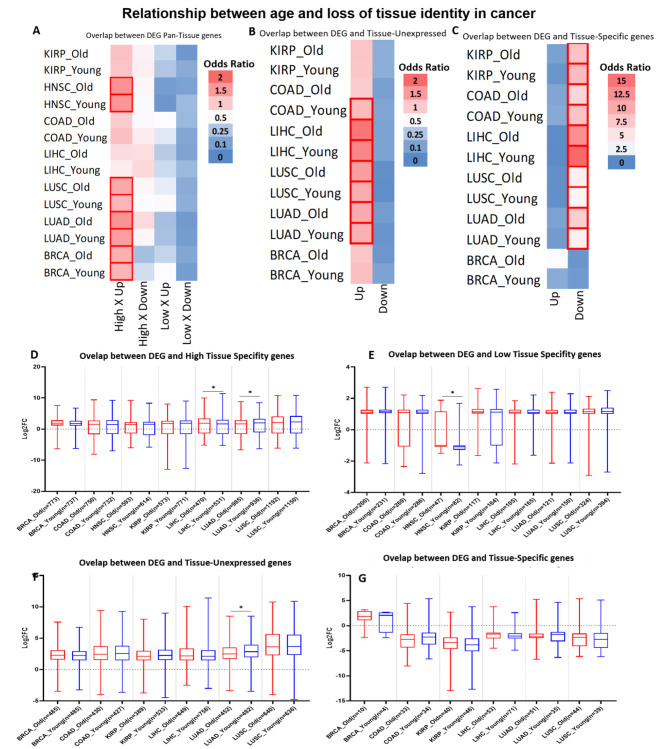
Overlap between cancer-DEGs and tissue specificity categories considering patient age groups. **A-C**, heatmap of odds ratio on the chance of the overlap. Red borders represent significant results (FDR < 0.05). **D-G**, plots comparing the expression level (Fold Change) of the overlap genes between DEGs and specificity categories in the two age groups (red = old and blue = young) significant results are represented with * (FDR < 0.05). Error bars represent the maximum and minimum values. Number of genes in each group is in parentheses on the x-axis. “Up” and “Down” represent whether genes are upregulated or downregulated. The “Old” is composed of DEGs resulting from the comparison between the 30% oldest cancer samples and the 30% oldest non-cancer tissue samples. The “Young” is obtained in a similar manner, but using the 30% youngest samples

Focusing on the main objective of this study, we repeated the overlap analysis as previously, but now considering the age groups (Fig. 4A-C). We can observe that the pattern is, in general, the same as previously observed, and age does not change it. Next, we directly compared the expression (fold change) of the genes of interest in relation to the four specificity categories (Fig. 4D-G). Although we have a few significant differences, they are slight and in the same direction, indicating that age is not significantly influencing the loss of cancer tissue identity, reinforcing this phenomenon may be essential for carcinogenesis.

## Discussion

Cell identity and plasticity are an essential topic in oncology and, more recently, are gaining importance in gerontology [9, 42, 43]. In this work, we analyze how changes in gene expression are related to tissue specificity during cancer and ageing, using data from thousands of human samples.

First, we sought to test the hypothesis that tissues lose their identity “naturally” in ageing. Although we see a trend of downregulation of Tissue-Specific genes (which could reinforce the hypothesis), it occurred in around 40%, suggesting that, at least, this phenomenon is not valid for the whole organism or is too subtle to be detected amid all the transcriptional noise in ageing, especially from bulk RNA-seq data [44]. Izgi et al., observed a loss of cellular identity in brain, lung, liver, and muscle in ageing mice, our results suggest similar findings only in brain, lung and partially in muscle [4]. In the same paper, the authors also analyzed GTEx data, and similar to our study, they did not find a clear pattern of inter-tissue convergence during ageing in humans. Interestingly, some tissues that lose their cellular identity are commonly affected by age-related diseases (brain with neurodegeneration, muscle with sarcopenia, prostate with benign prostatic hyperplasia, etc.), which may suggest a role of this dysfunction in pathologies [45–47]. However, further studies are needed to determine which cell types are affected, and the phenotypic consequences of identity loss before more robust conclusions can be drawn.

A question remains open: What mechanism leads to the loss of cellular identity observed in some tissues during ageing? One hypothesis is that these changes in the transcriptome are driven by age-related changes in the epigenome, a phenomenon known as epigenetic drift. Supporting this idea, we know that the epigenome is critical for maintaining cell identity, and epigenetic changes are associated with mammalian ageing [48–50]. Moreover, this epigenetic drift has been associated with age-related dysfunctions in both tissue-specific and non-specific ways, which shows that this hypothesis needs to be further studied in future studies [51]. It would also be interesting in the future to explore whether specific cell types in each tissue contribute to these patterns.

In cancer, on the other hand, we simultaneously observe an upregulation of High tissue specificity genes from other tissues, downregulation of Tissue-specific genes from the tissue of origin of the tumor, and activation of Tissue-unexpressed genes. This suggests that during tumorigenesis, cancer cells gain functions of other organs/tissues (or at least there are more upregulated genes because of the noise from generalized genome instability) while suppressing the functions of their original tissue. These results align with the literature since dedifferentiation is a known feature of cancer [52, 53]. This process has been described in some individual cancers such as colon, melanoma, and pancreas, but as far as we know, we were the first to demonstrate this in a pan-cancer analysis and in a tissue-specific manner [54–56].

Exploring cancer as a tissue-specific disease is an approach that is gaining prominence in oncology, with several studies trying to understand in depth the genetics that regulate this process [11, 57, 58]. Schaefera et al. explore why some genetic alterations are only relevant in specific types of cancer, concluding that the tissue microenvironment is a determining factor in this process [59]. Two other studies have demonstrated that expression signatures can help classify the tissue for cancers of unknown primary origin, which presents a possible application of using the transcriptome signatures with tissue specificity in oncology [60, 61]. Our work, besides adding novel knowledge to this field, corroborates studies such as that from Hu et al., which showed that in cancer, there is a decrease in the expression of some tissue-specific genes, and Pei et al., which showed that it is common for cancers to acquire specific expression profiles from other organs [62, 63].

When we directly compare cancer results with ageing, we have an interesting finding: in cancer, we have an upregulation of High tissue specificity genes, and in ageing a trend to downregulation. This kind of opposite pattern is expected and has already been described by our group [3]. But when analyzing the Tissue-Specific genes, most of the significant results are in the same direction, with the downregulation of these genes. This makes us wonder if preventing the loss of tissue specificity might be a promising strategy against cancer and ageing at the same time. But this result needs to be looked at carefully since less than half of the normal tissues show this pattern, and there are a few exceptions.

The pattern found also seems to influence the aggressiveness of cancer, impacting on patient survival. A phenomenon linked to the loss of tissue specificity is the acquisition of stemness and dedifferentiation, which is also related to the aggressiveness of cancers [53]. Furthermore, we still need better biomarkers in oncology, and although our data needs to be refined for possible application, tissue identity loss has the potential to improve the prognostic classification of cancer patients [64–66].

Finally, we tested the hypothesis that the age of patients influences expression patterns of specificity genes. We found no relevant difference between the young and old groups, indicating that age does not affect the process of tissue identity loss in cancer. This reinforces the newly proposed idea that the phenomenon of acquiring cellular plasticity (which includes loss of identity) is a hallmark of cancer [8].

In summary, we show evidence of a trend age-dependent loss of tissue specificity; however, this is not a global phenomenon, probably because it is more subtle in ageing. On the other hand, in cancer, we have a pattern of clear downregulation of Tissue-specific genes and activation of genes not expressed in the original tissue, including genes highly expressed in other tissues. Our results also suggest that this pattern influences cancer aggressiveness and is not influenced by the patient’s age, corroborating that it is a crucial step for carcinogenesis.

### Electronic supplementary material

Below is the link to the electronic supplementary material.

Supplementary Material 1

Supplementary Material 2

Supplementary Material 3

Supplementary Material 4

Supplementary Material 5

Supplementary Material 6

Supplementary Material 7

Supplementary Material 8

Supplementary Material 9

Supplementary Material 10

Supplementary Material 11

## Data Availability

The data underlying this article are available in the article and in its online supplementary material. The authors welcome readers to contact them for further questions and information. GTEx data is freely available at https://gtexportal.org/home/. TCGA data is freely available at https://www.cancer.gov/tcga.
