# Supplementary material for: Transcriptomic analysis reveals a tissue-specific loss of identity during ageing and cancer
Source: BMC Genomics. 2023 Oct 26;24:644. doi: 10.1186/s12864-023-09756-w (PMC10604446; doi:10.1186/s12864-023-09756-w)
Supplement: Supplementary file 11 — Supplementary Material 11 [file 12864_2023_9756_MOESM11_ESM.docx]

**Supplementary Materials**

**
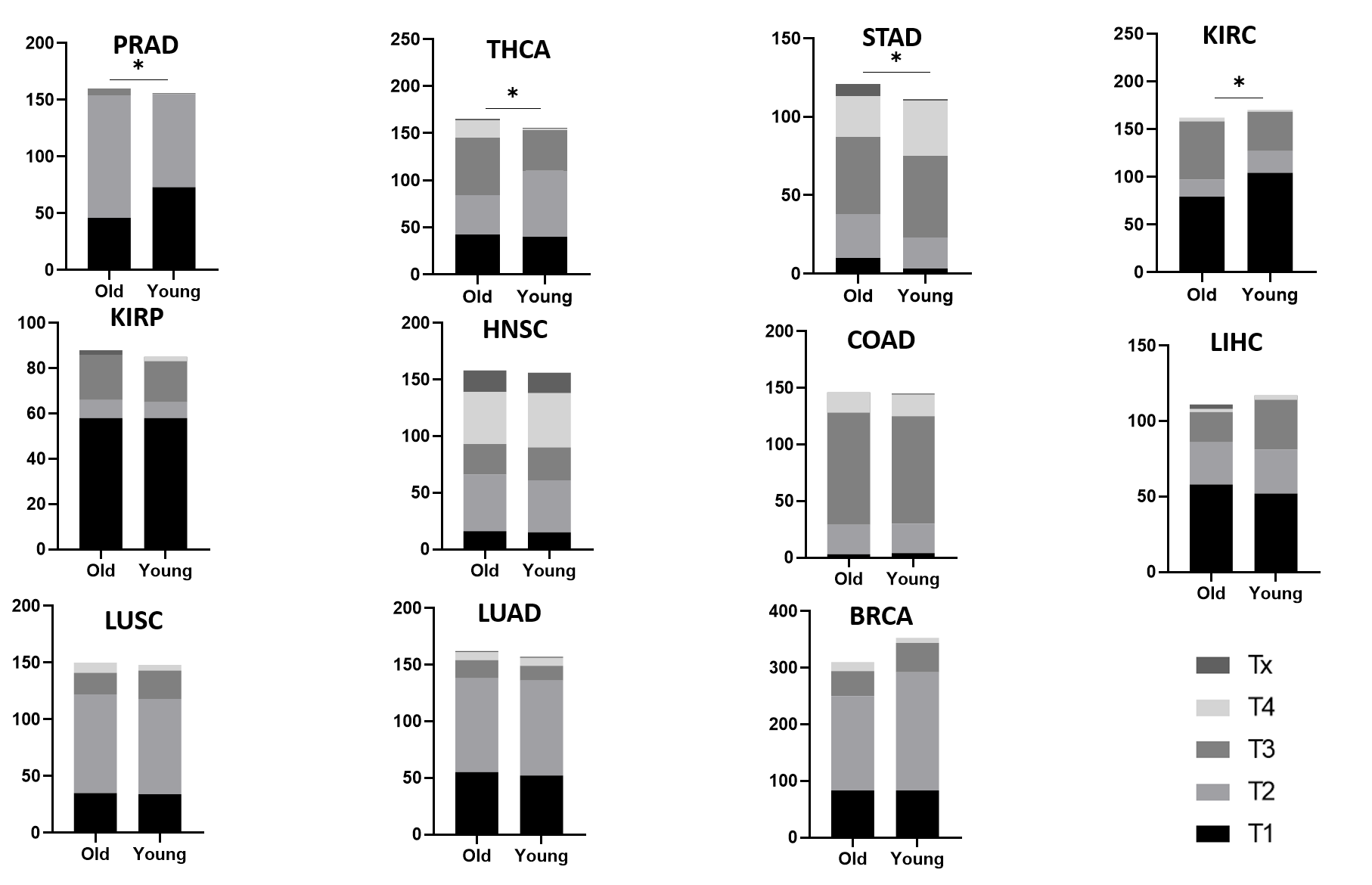
**

**Figure S1:** Comparison between the T pathological stage proportions of the old and young groups. The y-axis represents the number of patients. * p-value<0.05

**
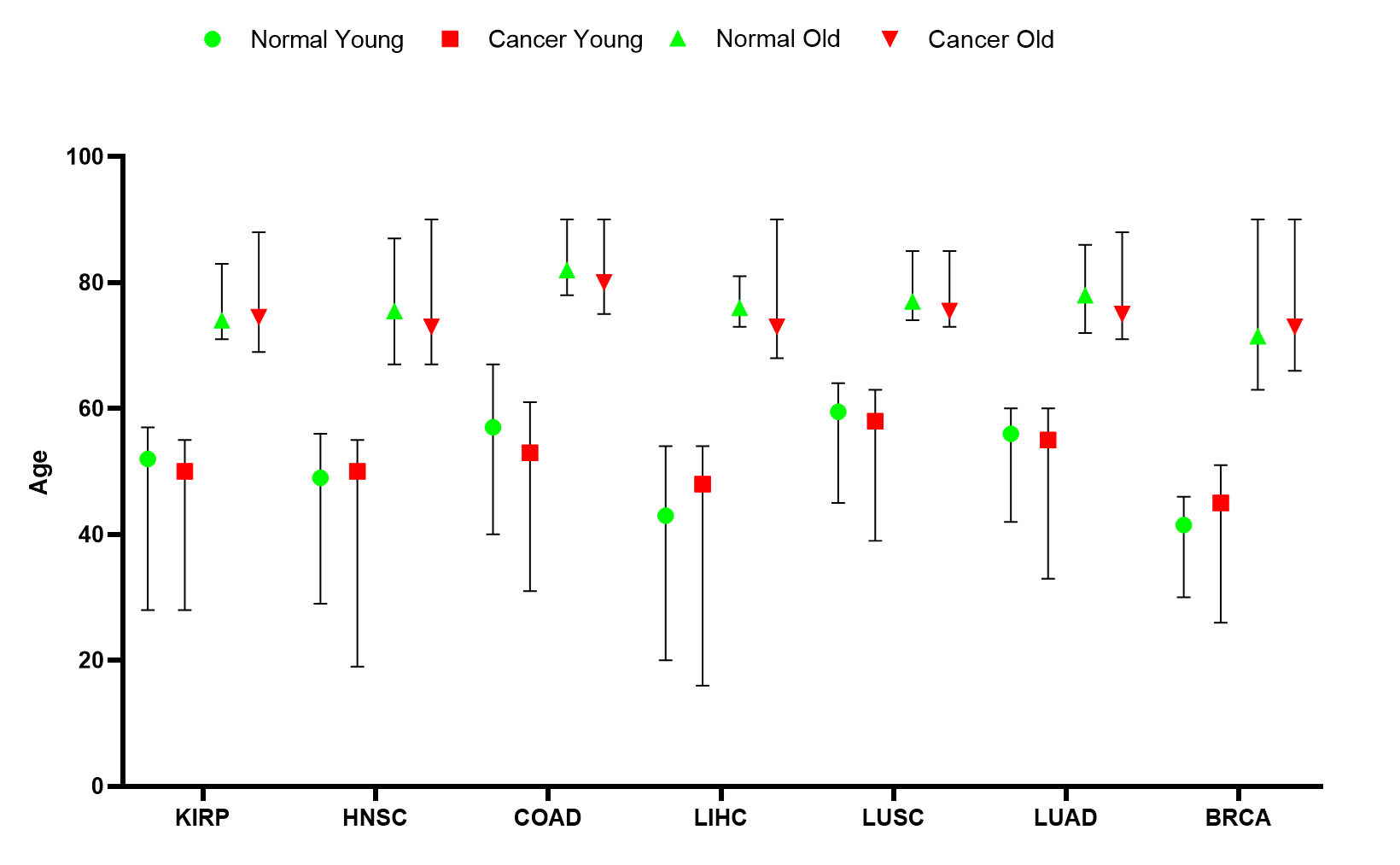
**

**Figure S2:** Age distribution of the seven cancers used in the age group analysis. Error bars represent the maximum and minimum values.

**
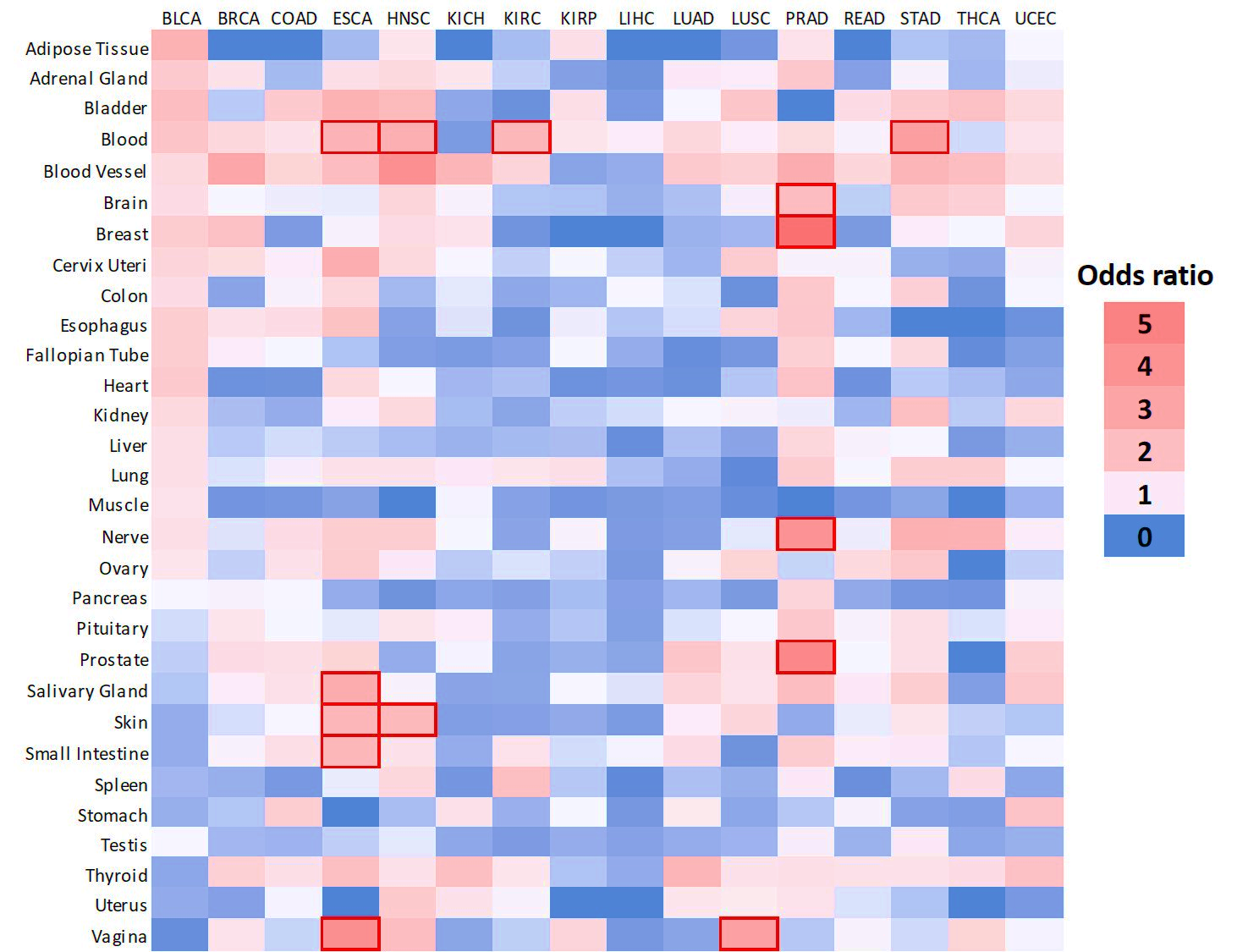
Figure S3: Overlap between upregulated cancer-DEGs and Tissue-Specific genes.** Heatmap of odds ratio on the chance of the overlap. In the columns we have the TCGA cancers and in the rows the GTEx tissues. Red borders represent significant results (FDR < 0.05).

**
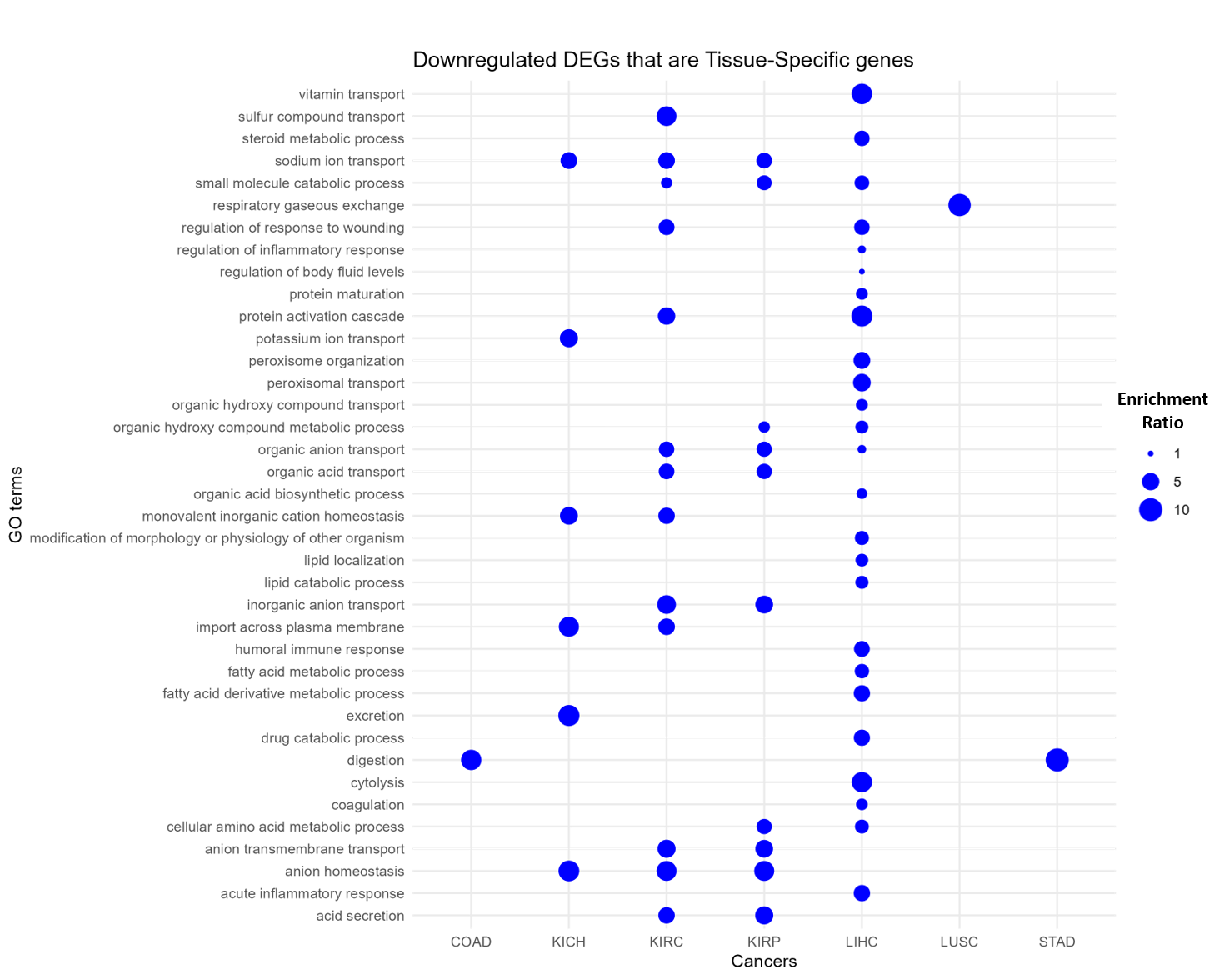
**

**Figure S4: Gene Enrichment Analysis.** Biological Processes (Gene Ontology) associated with downregulated Tissue-Specific cancer-DEGs. Only significant results are shown (FDR < 0.05). For better visualization, the enrichment ratio was log-transformed.

**
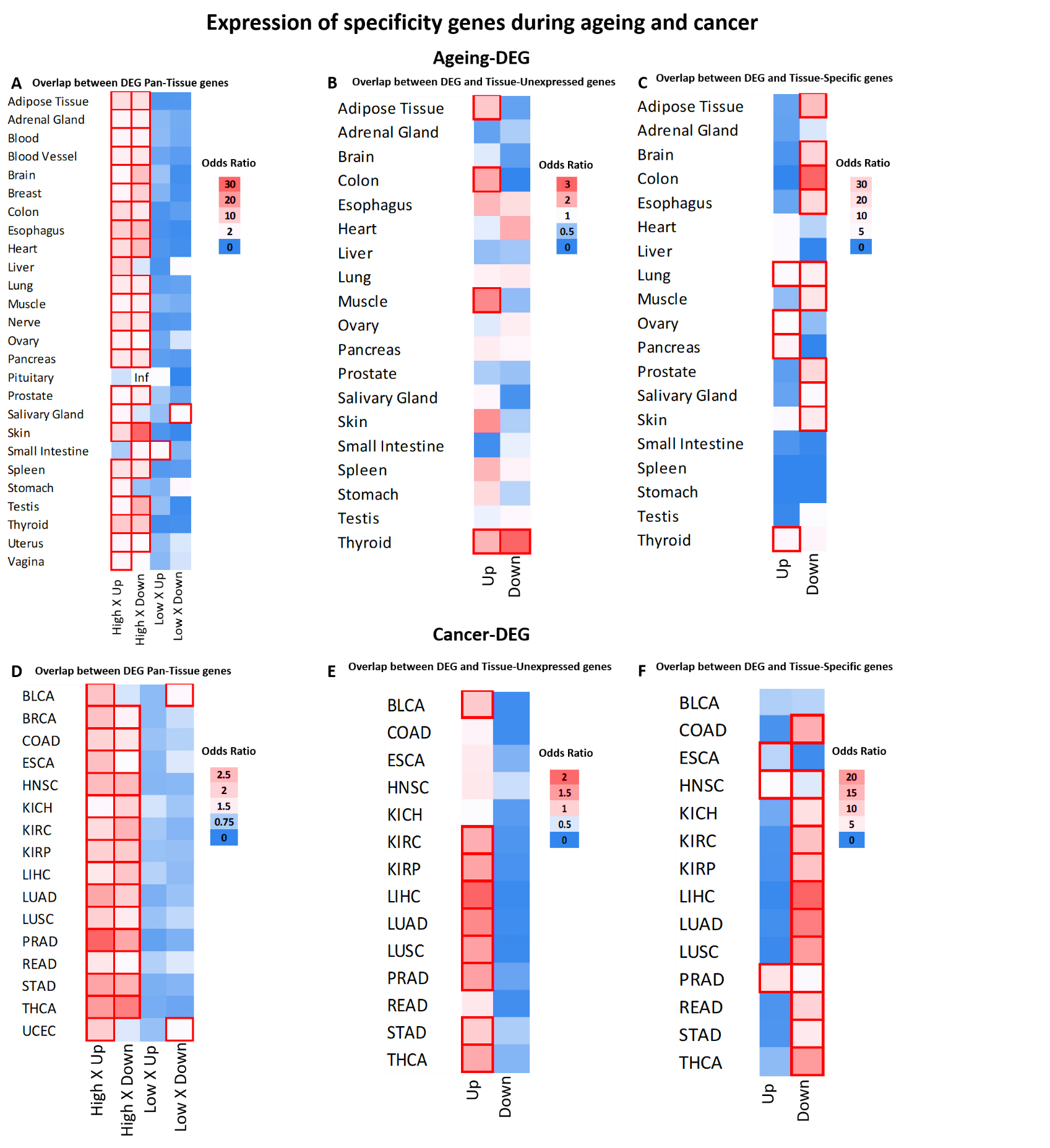
**

**Figure S5: Overlap between DEGs and alternative specificity categories.** Heatmap of odds ratio on the chance of the overlap. Red borders represent significant results (FDR < 0.05). A-C Ageing-DEGs, D-E Cancer-DEGs. Inf = Odds ratio tends to infinity due to the low number of downregulated DEGs, but the result is not significant. “Up” and “Down” represent whether genes are upregulated or downregulated.

**
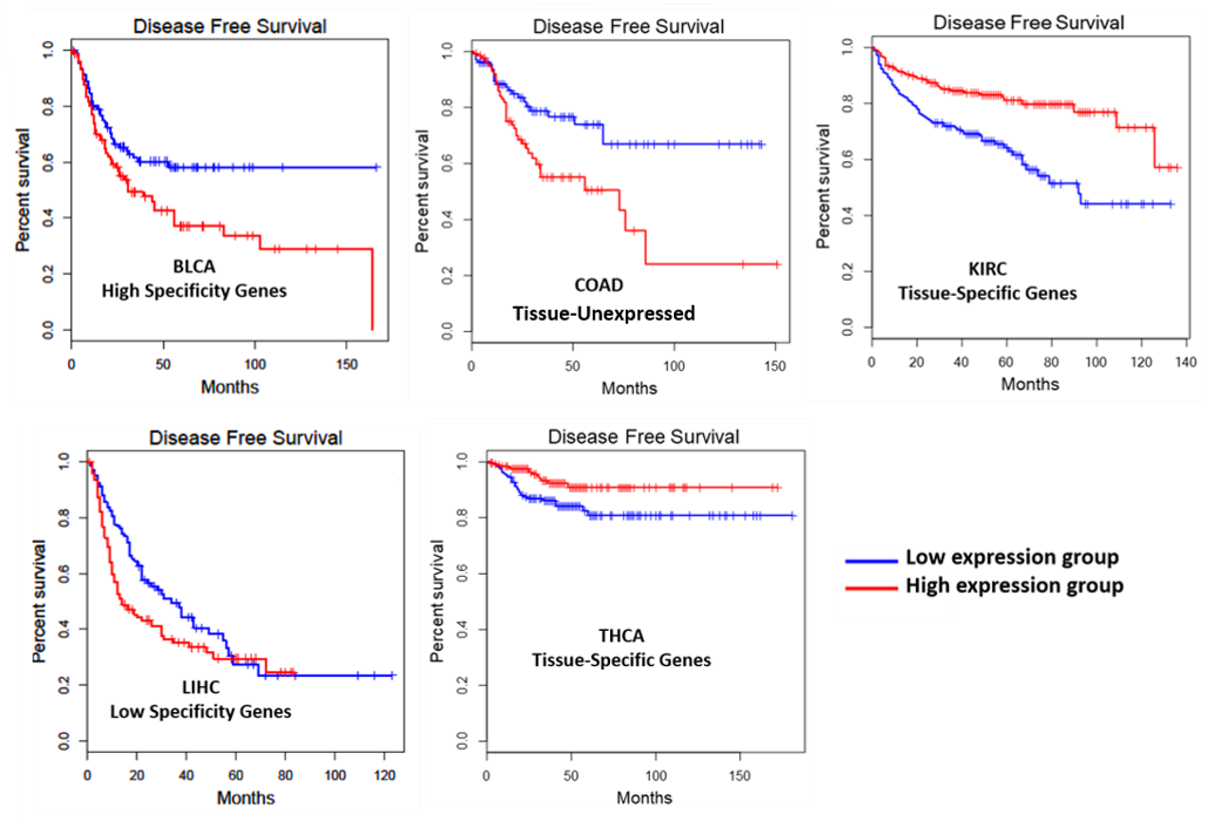
**

**Figure S6:** Kaplan-Meier curves of the significant disease free survival results from the Figure 3B.

**Supplementary tables:**

**Supplementary file 1 –** Number of Samples

**Supplementary file 2 –**Tau data

**Supplementary file 3 –** Alternative Category Genes

**Supplementary file 4 –** Cancer-DEGs

**Supplementary file 5 –** Ageing-DEGs

**Supplementary file 6 –** Genes in each tau’s categories

**Supplementary file 7–** Gene ontology of High and Low Tissue Specificity Genes

**Supplementary file 8 –** Gene ontology of upregulated Tissue-Unexpressed cancer-DEGs

**Supplementary file 9–** Cancer-DEGs old group

**Supplementary file 10 –** Cancer-DEGs young group
